# Enantioselective CE–MS analysis of ketamine metabolites in urine

**DOI:** 10.1002/elps.202200175

**Published:** 2022-12-09

**Authors:** Friederike A. Sandbaumhüter, Jordan T. Aerts, Regula Theurillat, Per E. Andrén, Wolfgang Thormann, Erik T. Jansson

**Affiliations:** ^1^ Department of Pharmaceutical Biosciences Uppsala University Uppsala Sweden; ^2^ Clinical Pharmacology Laboratory Institute for Infectious Diseases University of Bern Bern Switzerland; ^3^ Science for Life Laboratory Spatial Mass Spectrometry Uppsala University Uppsala Sweden

**Keywords:** capillary electrophoresis–mass spectrometry, chiral separation, hydroxynorketamine, ketamine, partial filling

## Abstract

The chiral drug ketamine has long‐lasting antidepressant effects with a fast onset and is also suitable to treat patients with therapy‐resistant depression. The metabolite hydroxynorketamine (HNK) plays an important role in the antidepressant mechanism of action. Hydroxylation at the cyclohexanone ring occurs at positions 4, 5, and 6 and produces a total of 12 stereoisomers. Among those, the four 6HNK stereoisomers have the strongest antidepressant effects. Capillary electrophoresis with highly sulfated γ‐cyclodextrin (CD) as a chiral selector in combination with mass spectrometry (MS) was used to develop a method for the enantioselective analysis of HNK stereoisomers with a special focus on the 6HNK stereoisomers. The partial filling approach was applied in order to avoid contamination of the MS with the chiral selector. Concentration of the chiral selector and the length of the separation zone were optimized. With 5% highly sulfated γ‐CD in 20 mM ammonium formate with 10% formic acid and a 75% filling the four 6HNK stereoisomers could be separated with a resolution between 0.79 and 3.17. The method was applied to analyze fractionated equine urine collected after a ketamine infusion and to screen the fractions as well as unfractionated urine for the parent drug ketamine and other metabolites, including norketamine and dehydronorketamine.

**Abbreviations**
DHK: dehydroketamine
DHNK: 5,6‐dehydronorketamine
HK: hydroxyketamine
HNK: hydroxynorketamine
KET: ketamine
MNK: methoxynorketamine
NK: norketamine

## 1 INTRODUCTION

Advantages of the racemic drug ketamine (KET) over classical antidepressants such as selective serotonin reuptake inhibitors are the rapid onset, long‐lasting effects, and robust efficacy, in particular, in therapy‐resistant patients. The molecular mechanism behind the antidepressant effects of the drug that is widely used as an *N*‐methyl‐d‐aspartate receptor antagonist for anesthesia and analgesia remains unclear [1, 2, 3, 4, 5, 6, 7]. Hydroxynorketamine (HNK) stereoisomers are metabolites of KET and have become a focus of interest for drug repurposing, as they have shown strong antidepressant effects, whereas typical side effects known from the treatment with KET, such as dissociation and substance abuse, were absent [4, 8, 9]. Preclinical studies have revealed the (2*R*,6*R*)‐6HNK enantiomer as having the strongest antidepressant effect followed by (2*S*,6*R*)‐6HNK, (2*R*,6*S*)‐6HNK, and (2*S*,6*S*)‐6HNK [8].

**FIGURE 1:**
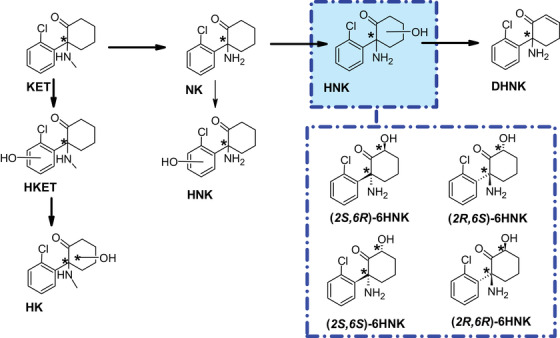
Metabolic pathway of ketamine (KET). In the main pathway KET is first *N*‐demethylated to norketamine (NK), that is, further hydroxylated and dehydrogenated. Other metabolites are hydroxyketamine (HK) and hydroxynorketamine (HNK) with the hydroxylation at the chlorophenyl ring. The four 6HNK stereoisomers showed antidepressant effects. Chiral centers are marked with “*.”

These enantiomers arise when KET is stereoselectively metabolized by cytochrome P450 enzymes. The main metabolic pathway leads KET first via *N*‐demethylation to norketamine (NK), next followed by hydroxylation at the cyclohexanone ring to HNK and finally to 5,6‐dehydronorketamine (DHNK) (Figure 1). With the hydroxylation at the cyclohexanone ring, a second chiral center is introduced; thus, different stereoisomers can be formed [1, 4, 10–16]. In addition, some minor metabolites are hydroxyketamine (HK), NK stereoisomers with a hydroxylation at the chlorophenyl ring, and an unstable *N*‐oxide [1, 4, 10–18].

To better understand the antidepressant effects of KET and the role of the pharmacologically active metabolites, sensitive, enantioselective, and robust analytical methods covering all KET metabolites are required. Mass spectrometry (MS) detection provides great sensitivity, selectivity, and multiplexing capabilities, which allows for detailed analysis without need for chemical labeling. Current methods that couple MS with liquid chromatography (LC) or supercritical‐fluid chromatography (SFC) lack either stereoselectivity completely or cover only the parent drug and the main metabolites NK, DHNK, and 6HNK [6, 18, 19–21]. In contrast to LC and SFC that require enantioselective stationary columns, capillary electrophoresis (CE) has the possibility of having chiral selectors in the background electrolyte (BGE), which in turn offers a high degree of flexibility. Various bioanalytical assays based on CE coupled to an ultraviolet light detector (CE–UV) using sulfated cyclodextrin (CD) as a chiral selector have been developed for the analysis of KET and its metabolites [10, 14, 17, 22–25]. With a mixture of sulfated β‐CD and highly sulfated γ‐CD, it was possible to separate eight HNK stereoisomers present in horse urine after KET infusion [17]. One caveat with this method is that sulfated CDs as sodium salts are not suitable for MS, because they cause suppression effects [26]. The partial filling approach originally developed to overcome disturbances caused by UV‐absorbing material in CE–UV setups was later applied in CE–MS to prevent chiral selectors from fouling the MS [27, 28, 29, 30]. Thus, besides enantioselective qualitative and quantitative analysis of analytes of interest in complex biological samples, also structural information for metabolite identification can be collected. Initial applications of the partial filling approach combined with MS include the enantioselective analysis of tetrahydroberberine, tetrahydropalmatine, amphetamine derivatives, ibuprofen and metabolites, native and derivatized amino acids as well as dipeptides [27, 30–34].

Here, we present the first enantioselective CE–MS method for a simultaneous analysis of HNK stereoisomers with specific focus on the four 6HNK stereoisomers. Our method is based on a partial filling approach, using negatively charged, and highly sulfated γ‐CD as a chiral selector. We deployed our method to screen for KET metabolites, including several HK stereoisomers in unfractionated and fractionated pony urine collected after administration of racemic KET.

## 2 MATERIALS AND METHODS

### 2.1 Chemicals, reagents, and origin of equine urine

KET as Ketador vet. (100 mg/ml) was from Richter Pharma (Vienna, Austria). NK (as hydrochloride in methanol, 1 mg/ml of the free base) and DHNK (as hydrochloride in acetonitrile, 100 µg/ml) were purchased from Cerilliant (Round Rock, TX, USA). Analytical standards of (2*S*,6*S*)‐6HNK and (2*R*,6*R*)‐6HNK were from Dr. Irving Wainer (Laboratory of Clinical Investigations, National Institute on Aging, National Institutes of Health, Baltimore, MD, USA). Standards for (2*S*,5*S*)‐5HNK, (2*R*,5*R*)‐5HNK, (2*S*,5*R*)‐5HNK, (2*R*,5*S*)‐5HNK, (2*S*,4*S*)‐4HNK, (2*R*,4*R*)‐4HNK, (2*S*,4*R*)‐4HNK, and (2*R*,4*S*)‐4HNK as well as (2*S*,6*R*)‐6HNK and (2*R*,6*S*)‐6HNK were synthetized according to Morris et al. [35] and kindly provided by the Division of Preclinical Innovation, NCATS, National Institutes of Health (Rockville, MD, USA). Highly sulfated γ‐CD (20% w/v solution) was from Beckman Coulter (Fullerton, CA, USA). Sulfated β‐CD (lot 04426HJ), sodium hydroxide flakes 97%, and dichloromethane were from Sigma‐Aldrich (St. Louis, MO, USA). Optima water, formic acid, and methanol came from Fisher Scientific (Göteborg, Sweden). Mixtures of the standard compounds were prepared in water, methanol, and acetic acid (50:49.5:0.5).

The equine urine was from a healthy Shetland pony of a previously described KET target‐controlled infusion study under isoflurane in oxygen anesthesia and was approved by the committee for animal experimentation of Kanton Bern, Switzerland [36]. To obtain samples with HNK, the urine was fractionated using HPLC as described previously [14]. After hydrolysis with β‐glucuronidase/arylsulfatase, analytes were extracted at alkaline pH with dichloromethane/ethyl acetate (75:25% v/v). The sample was dried and reconstituted in water prior to injection onto a Purospher RP 18e column (4 mm × 125 mm × 5 µm) (Merck, Darmstadt, Germany) installed on a Waters LC‐Module I plus with absorbance detector (Waters Corporation, Milford, MA, USA). Hydroxylated NK metabolites were separated and collected in four fractions (I–IV) [14]. Urine and collected fractions were stored at −20°C until further use.

### 2.2 Sample preparation

Samples were prepared as described previously [14]. Briefly, to 50 µl of an equine urine sample (urine fraction, plain urine) 200 µl water, 50 µl 0.5 M NaOH, and 1300 µl dichloromethane were added. After mixing (10 min) and centrifugation (13 000 rpm, 5 min), the aqueous phase was removed. A total of 10 µl of 1 mM acetic acid were added to the organic phase, and the sample was dried in an Eppendorf concentrator 5301 (Hamburg, Germany) at 45°C. The sample was reconstituted in a mixture of water, methanol, and acetic acid (50:49.5:0.5).

### 2.3 CE–MS

**FIGURE 2:**
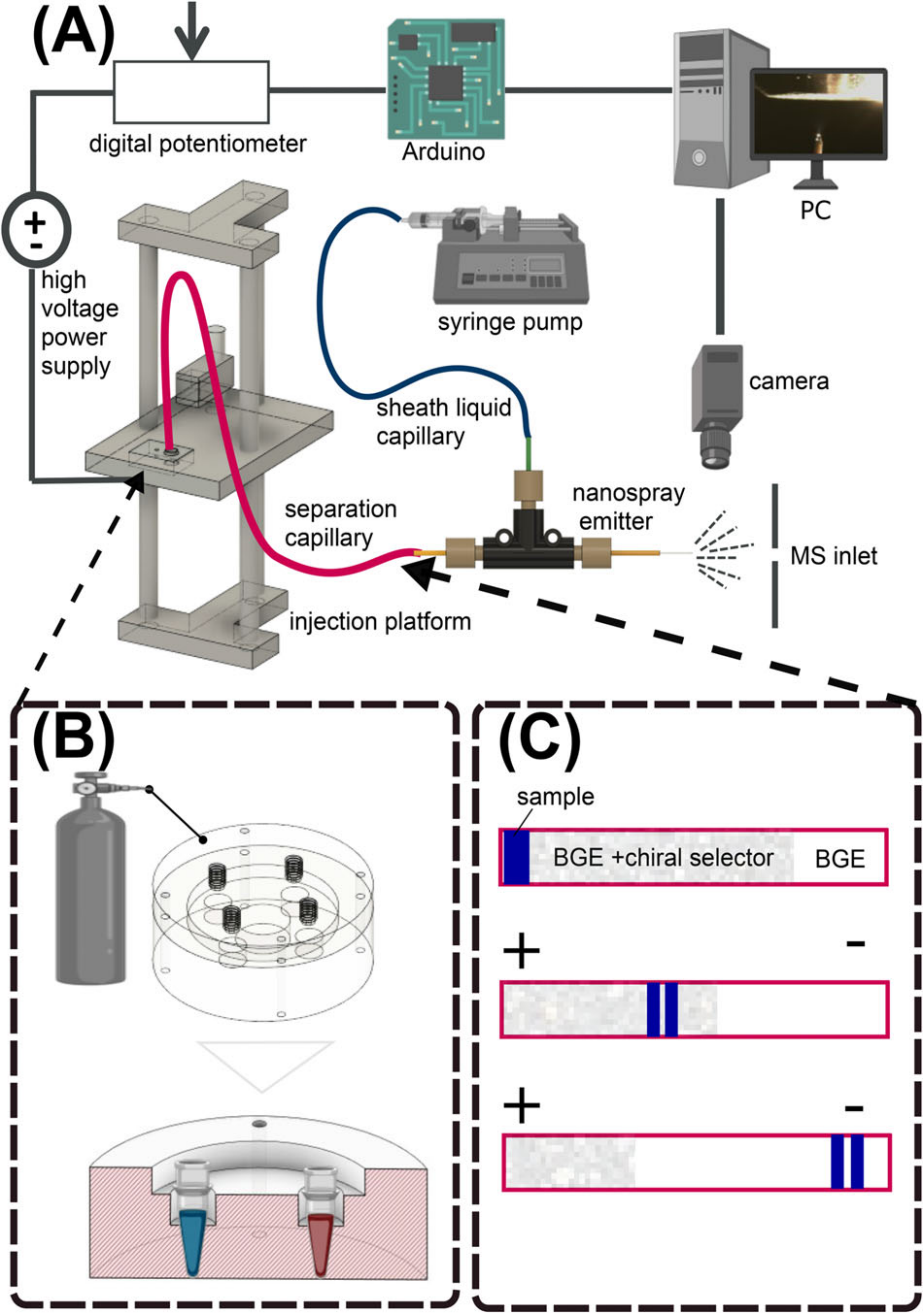
(A) Overview of the capillary electrophoresis–mass spectrometry (CE–MS) setup. The sample is injected from a vial on the injection platform into the separation capillary by lifting the platform. The platform is coupled to a high‐voltage power supply operated by using a digital potentiometer. The analytes are introduced to the MS via a nanospray emitter. For spray stability sheath liquid is used. The spray stability is monitored with a camera. (B) Before and between analytic runs, the separation capillary is flushed with water, NaOH, and background electrolyte (BGE) using a pressurized vial holder. (C) Partial filling was used for the chiral separation. A plug containing BGE without a chiral selector was pushed to the end of the capillary. The separation zone consisted of BGE with a chiral selector. Last the sample plug was injected. When voltage was applied the analytes became transported toward the mass analyzer. Chiral separation took place in the presence of the chiral selector.

CE separations were performed and gas phase ions generated using a coaxial sheath flow CE–electrospray ionization (ESI) interface previously described by Nemes et al. [37] (Figure 2A).

#### 2.3.1 CE

Direct current high‐voltage power supply (HVPS) model HPS100‐40‐0.4 (Beijing Excellent Innovate HD Electronics Co., Ltd, Beijing, China) was operated in remote configuration using an MCP41HV51‐103E/ST digital potentiometer controlled by an Arduino Uno Rev3 (Arduino SA, Sommerville, MA, USA) with code written in Arduino IDE to reproducibly ramp the HVPS over 60 s to the separation potentials of 15–26 kV. Separations were performed in an anode‐to‐cathode mode in a 50–100 cm fused‐silica separation capillary with an inner diameter of 40 µm and an outer diameter of 140 µm (Trajan Scientific and Medical, Victoria, Australia) or 105 µm (Polymicro Technologies, Phoenix, AZ, USA). Hydrodynamic sample injection volumes ranged from 19 to 37 nl. The electrical circuit was connected to earth ground via the stainless‐steel needle of the sheath liquid syringe. The separation capillary was conditioned between injections with ∼3 column volumes H_2_O, ∼3 column volumes 0.1 M NaOH, ∼5 column volumes H_2_O, followed by ∼3 column volumes of BGE (10% formic acid, 20 mM ammonium formate) delivered at 20 psi of house nitrogen by connecting the separation capillary inlet to a laboratory built acrylic holder for solution vials using P‐683‐1/4‐28 male to luer lock assemblies (IDEX Health and Science, Oak Harbor, WA, USA) (Figure 2B). Partial filling with chiral selector was performed in the same manner with 20 psi house nitrogen using filling times based on the measurement of sodium formate clusters pushed through the capillary (Figure 2C).

#### 2.3.2 Electrospray ionization

The model of PEEK tee used was P‐727 and attached to a piece of optical breadboard fastened to an FSL40XYZ‐L linear ball screw gantry stage controlled by AMC4030 motion control software (both Fuyu Technology, Chengdu, China) to position the ESI emitter at the mass spectrometer inlet. Stainless‐steel hypodermic tubing, 270 µm outer diameter, 160 µm inner diameter (G. Kinnvall AB, Sparreholm, Sweden) was used for the ESI emitter assembly, 1.5–2.5 kV electrospray voltage was applied directly to the stainless‐steel tubing and controlled using MassLynx software (Waters Corporation). The sheath liquid was composed of 60% methanol and 0.1% formic acid and was applied at a flow rate of 500 nl/min.

#### 2.3.3 Mass spectrometry

Generated ions were analyzed by QTOF‐MS on a Synapt G2Si (Waters Corporation) operated in positive ion mode within a mass range of 50–500 Da. The instrument was calibrated on the day of use using a 20 psi infusion of 0.01 M NaOH through the separation capillary to generate sodium formate clusters when reaching the capillary outlet and mixing with the sheath liquid.

## 3 RESULTS AND DISCUSSION

### 3.1 Method development

#### 3.1.1 BGE and chiral selector

The chemical composition, pH, and the ionic strength of the BGE affect the separation performance. In the case of chiral separation, the type and concentration of the chiral selector also play an important role. Three different BGE compositions, 5% acetic acid, 20 mM ammonium formate with 10% formic acid, and 1% formic acid, were tested. These were each followed up with various concentrations of highly sulfated γ‐CD, sulfated β‐CD, and mixtures of the two preparations as described below. The selected CDs were previously successfully applied in different assays to achieve chiral separation of KET and its metabolites [10, 14, 17, 22, 23, 24, 25]. Different concentrations were tested because small changes in CD concentrations can have a critical impact on the chiral separation especially for closely related compounds such as the HNK stereoisomers [17, 24, 38]. The results are discussed in more detail in the next section.

We found that 20 mM ammonium formate with 10% formic acid showed the most stable results. No differences in the performance of sulfated β‐CD and highly sulfated γ‐CD were found. The same was true for a mixture of β‐ and γ‐CD that successfully separated the HNK stereoisomers in a CE–UV method [17]. Based on reports about high batch variability of sulfated β‐CD (7–11 mol sulfate/mol CD) that affected the performance as a chiral selector and could require a method modification for each batch, we continued with less variable highly sulfated γ‐CD (12–13 mol sulfate/mol CD) [38]. Further, we observed that coating of the inner capillary wall with linear polyacrylamide as it was reported for the chiral separation of basic compounds in the partial filling CE–MS method of Yan et al. [27] did not have any advantages over using bare fused‐silica capillaries.

#### 3.1.2 CD concentration and plug length

Combining the advantages of CE and MS for chiral separations requires measures to avoid contamination of the MS with the chiral selector. Sulfated and highly sulfated CDs that were used as chiral selectors in this work are negatively charged and migrate away from the MS inlet when the latter is acting as the cathode of the CE circuit. This is also known as countercurrent migration and was successfully applied for the enantioselective separation of various drugs and their metabolites [31, 39]. In our assay, a plug containing BGE without CD was placed at the anodic end of the capillary and the remaining part was filled with BGE containing CD (separation zone) (top graph of Figure 2C). After sample injection and application of separation potential, the CD begins to migrate toward the anode into and through the sample zone and analytes interact with the selector.

The partial filling was tested by infusing 0.1 M sodium formate in water into the separation capillary. The time it took to detect sodium formate clusters in the mass spectrum was determined and used for timing the injection of the different zones. The filling time was recorded for each capillary.

Both CD concentration and plug length affected the separation and were optimized in order to achieve chiral separation of the analytes of interest. A mixture of *S*‐ and *R*‐KET, *S*‐ and *R*‐NK, and (2*R*,6*S*)‐ and (2*S*,6*R*)‐6HNK was injected as the sample. Ammonium formate with a concentration of 20 mM with 10% formic acid was used as BGE. Highly sulfated γ‐CD in concentrations between 0% and 5%, sulfated β‐CD (1% and 2%), and mixtures of the two preparations (0.5% each and 1% each) were tested as well as fillings of 30% and 75%.

At CD concentrations larger than a compound specific level (e.g., at concentrations > ∼0.8% and > ∼1.4% of highly sulfated γ‐CD for KET and NK, respectively [40]) the analytes migrate in the anionic direction. KET with the strongest interaction with the chiral selector moves the fastest followed by NK and HNK [22, 23]. Consequently, in the developed assay format with the anionic migration of the chiral selector, HNK spends the least amount of time in contact with the selector.

The length of the CD plug determines how long the chiral selector can interact with the analytes. With 30% filling, almost no change was found compared to injections into a BGE without the chiral selector. Increasing the filling to 75% led to longer migration times but provides more time for interaction between analytes and the chiral selector. The consequence is a separation of the HNK analytes that could be further improved by a variation of the chiral selector concentration (Table 1).

**TABLE 1:** Migration times of (2*R*,6*S*)‐ and (2*S*,6*R*)‐6HNK and their resolution using different concentrations of highly sulfated γ‐cyclodextrin (CD) and sulfated β‐CD^a^

Concentration cyclodextrin (%)		Partial filling (%)	Migration time (min)		Resolution
*γ*	*β*		(2*R*,6*S*)‐6HNK	(2*S*,6*R*)‐6HNK	
0	0	None	38.3	38.3	None
0.5	0	75	51.5	51.8	0.86
1	0	75	57.1	57.7	0.93
2	0	75	64.5	64.9	1.03
5	0	75	88.5	91.3	3.74
0	1	75	35.7	36.2	1.39
0	2	75	51.3	52.7	1.81

^a^ A bare‐fused‐silica capillary (length between 93 and 100 cm; 40 µm inner diameter) was employed. The voltage was adjusted so that a stable current and spray was obtained and varied between 17 and 25 kV. The background electrolyte contained 20 mM ammonium formate and 10% formic acid (pH 1.68).

**FIGURE 3:**
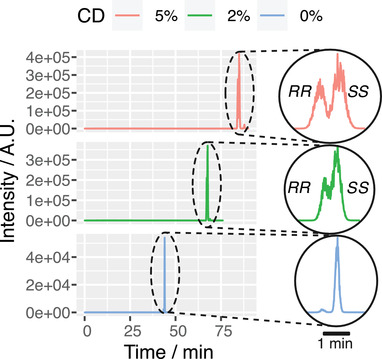
Urine fraction I was observed to contain (2*R*,6*R*)‐6HNK and (2*S*,6*S*)‐6HNK. The fraction was analyzed using 0%, 2%, and 5% γ‐CD in the partial filling approach with 75% filling. By increasing the CD concentration, the migration times increase and the separation improves.

With 2% highly sulfated γ‐CD and 75% filling, the migration times (*n* = 6) of (2*R*,6*S*)‐6HNK and (2*S*,6*R*)‐6HNK were 63.8 ± 1.7 min (mean ± SD) and 64.5 ± 1.6 min, respectively. The relative standard deviation was 2.6% and 2.5%, respectively. Increasing the CD concentration to 5% led to longer migration times but also improved the resolution (*R*) of (2*R*,6*S*)‐6HNK and (2*S*,6*R*)‐6HNK from 0.86 (0.5% CD) to 3.74 (5% CD) (Table 1). The same was shown for the analysis of fractionated horse urine. With increasing γ‐CD concentration the separation of the enantiomers (2*R*,6*R*)‐6HNK and (2*S*,6*S*)‐6HNK improved (Figure 3). *R* was calculated as

$$R=1.175\frac{MT_{2}-MT_{1}}{FWHM_{1}+FWHM_{2}}$$

where *MT*_2_ and *MT*_1_ are the migration times of the second and the first peak, respectively, and *FWHM*_1_ and *FWHM*_2_ give the full width at half maximum for peak 1 and 2, respectively. *R* of ≥1.5 ranks as baseline separation [38].

With 5% highly sulfated γ‐CD, no chiral separation of KET, NK, and DHNK was observed. Strongly interacting analytes, such as KET and NK, become lost in the absence of a fluid flow toward the cathode. As observed previously in the development of a CE–UV method, it was not possible to analyze simultaneously the enantiomers of KET, NK, DHNK, and the HNK stereoisomers within an acceptable run time. Therefore, we focused on the HNK stereoisomers that are of interest for the development of antidepressant therapies and for which there is a lack of analytical methods. As they have a weaker interaction with the chiral selector [22, 23], they migrate more slowly in the anodic direction, become separated, and are presumably concomitantly pushed toward the cathode by a small cathodic buffer flow caused by residual electroosmotic flow and fluid siphoning from the sheath liquid interface (center graph of Figure 2C). Eventually, the negatively charged CD migrates away from the analytes and the separated stereoisomers are transported by the combined action of cationic electromigration in the CD‐free zone and the residual fluid flow to the MS (bottom graph of Figure 2C).

For stereoselective analysis of HNK stereoisomers, we used a BGE composed of 20 mM ammonium formate with 10% formic acid and 5% highly sulfated γ‐CD (pH 1.68) and 75% filling. Compared to currently available CE–UV assays, this method combining CE and MS provides a greater selectivity and allows for a simultaneous screening of other metabolites arising from KET and tentative co‐medication.

### 3.2 Analysis of HNK stereoisomers

**FIGURE 4:**
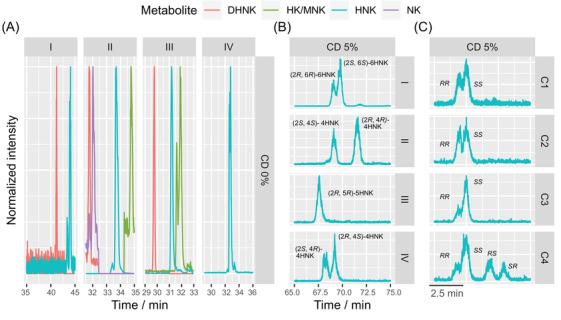
(A) The four urine fractions were screened without the chiral selector for the masses of the metabolites norketamine (NK; *m*/*z* = 224.08), 5,6‐dehydronorketamine (DHNK; *m*/*z* = 222.069), hydroxynorketamine (HNK; *m*/*z* = 240.07), and hydroxyketamine /methoxynorketamine (HK/MNK; *m*/*z* = 254.09). Shifts in migration times are due to differences in separation potential between fractions. (B) Electropherograms for HNK (*m*/*z* = 240.07) of all four urine fractions. Fractions I, II, and IV contain two HNK peaks each, whereas fraction III has only one. (C) Electropherograms of the 6HNK stereoisomers that are of pharmacological interest. (C1) A 10x dilution of fraction I, observed to contain (2*S*,6*S*)‐6HNK and (2*R*,6*R*)‐6HNK. (C2) Sample C1 spiked with (2*R*,6*R*)‐6HNK. (C3) Sample C1 spiked with (2*S*,6*S*)‐6HNK. (C4) Sample C3 spiked with (2*R*,6*S*)‐6HNK and (2*S*,6*R*)‐6HNK. For panels (B) and (C), 5% of highly sulfated cyclodextrin (CD) was employed for separation, whereas none was used in panel (A).

Equine urine obtained after a KET target‐controlled infusion under isoflurane in oxygen anesthesia was extracted and fractionated with HPLC on an RP‐C18 column. As KET metabolism is stereoselective, not all theoretically possible metabolites and stereoisomers are formed *in vivo*. In general, 80% of the KET‐related material in the urine is HNK and glucuronidated HNK, respectively [41]. Unfractionated urine and four urine fractions that contained HNK stereoisomers according to previous experiments [14] were analyzed with our CE–MS method. First, we performed the analysis without a chiral selector (Figure 4A). In the absence of sulfated CD, all compounds migrate as cations toward and into the ESI‐MS interface and we screened for the masses corresponding to KET (*m*/*z* = 238.09), NK (*m*/*z* = 224.08), HNK (*m*/*z* = 240.07), DHNK (*m*/*z* = 222.069), dehydroketamine (DHK, *m*/*z* = 236.08), as well as to HK (*m*/*z* = 254.09) and methoxynorketamine (MNK, *m*/*z* = 254.09). DHK, DHNK, NK, KET, and HNK were detected in the unhydrolyzed urine. DHNK and HNK were found in fraction I, DHNK, HNK, NK, and HK/MNK in fraction II, DHNK, HNK, and HK/MNK in fraction III, and HNK in fraction IV (Figure 4A). The identity of the peaks was confirmed by experiments with standard compounds.

HK and MNK are isobars both with two chiral centers. Thus, the formation of several stereoisomers is theoretically possible. Comparing analyses without CD and 2% highly sulfated γ‐CD shows the presence of different stereoisomers with an *m*/*z* of 254.09. Due to the lack of analytical standards and of urine collected after treatment with only one KET enantiomer, no further assignment is possible. Nevertheless, it shows the great potential of this method to provide deeper metabolic information than with the CE–UV assay where a separation of every single peak is necessary.

Second, we analyzed the four equine urine fractions using the partial filling approach with 75% filling and 5% highly sulfated γ‐CD. As expected, HNK was found in all fractions (Figure 4B). This agrees with the findings of Schmitz et al. who analyzed equine urine fractions collected according to the same protocol with LC–MS and with chiral CE–UV [14]. Using the partial filling approach with highly sulfated γ‐CD as a chiral selector resulted in two HNK peaks per fraction except fraction III. Fraction I was previously identified as (2*S*,6*S*)‐6HNK and (2*R*,6*R*)‐6HNK by spiking it with analytical standards [17]. In the same way the peaks in fraction II could now be assigned to (2*S*,4*S*)‐4HNK and (2*R*,4*R*)‐4HNK, those of fraction III to (2*S*,5*S*)‐5HNK and (2*R*,5*R*)‐5HNK, and those in fraction IV to (2*S*,4*R*)‐4HNK and (2*R*,4*S*)‐4HNK (data not shown). In each fraction, there was a difference in the abundance of the enantiomers indicating the stereoselectivity in the metabolism. More (2*S*,6*S*)‐6HNK than (2*R*,6*R*)‐6HNK was formed. The same was found previously in mice [8]. In human plasma collected after treatment with KET and then analyzed with an achiral LC–MS method, 6HNK was described in the literature as the major circulating metabolite ((2*S*,6*R*); (2*R*,6*S*)‐6HNK > (2*S*,6*S*); (2*R*,6*R*)‐6HNK) followed by (2*S*,5*S*); (2*R*,5*R*)‐5HNK, (2*S*,4*R*); (2*R*,4*S*)‐4HNK, (2*S*,4*S*); (2*R*,4*R*)‐4HNK, and (2*S*,5*R*); (2*R*,5*S*)‐5HNK. In contrast, in human urine (2*S*,5*R*); (2*R*,5*S*)‐5HNK was dominant followed by (2*S*,6*S);* (2*R*,6*R*)‐6HNK, (2*S*,5*S*); (2*R*,5*R*)‐5HNK, (2*S*,6*R*); (2*R*,6*S*)‐6HNK, (2*S*,4*R*); (2*R*,4*S*)‐4HNK, and (2*S*,4*S*); and (2*R*,4*R*)‐4HNK [12, 18]. We further found that (2*R*,4*S*)‐4HNK was more abundant than (2*S*,4*R*)‐4HNK and there was more (2*R*,4*R*)‐4HNK than (2*S*,4*S*)‐4HNK.

Furthermore, we searched for the known fragment *m*/*z* = 125; a chloromethylbenzene fragment derived from either KET, NK, DHNK, HNK, or HK [21, 42]. Fragmentation information was gained through in‐source fragmentation processes. This fragment was found in all four urine fractions that confirmed the hydroxylation at the cyclohexanone ring. The hydroxylation at the chlorophenyl ring would lead to a fragment of *m*/*z* = 141 [14, 42]. Another fragment originating from HNK is *m*/*z* = 222 that was also present in all fractions. The *m*/*z* = 222 traces for fractions I, II, and III additionally showed a peak at the migration time of DHNK (*m*/*z* of 222). The formation of the double bond between positions 5 and 6 in the cyclohexanone ring yields that DHNK could arise from a loss of the OH‐group at position 6 in 6HNK and 5 in 5HNK. Consequently, DHNK is present in the fractions containing 6HNK and 5HNK (fractions I and III, respectively). We further detected DHNK and NK in fraction II. These two compounds where not found by Schmitz et al. [14].

Spiking fraction I with a mixture of all 6HNK stereoisomers confirmed not only the identity of the two peaks of fraction I as (2*S*,6*S*)‐6HNK and (2*R*,6*R*)‐6HNK but also showed the capability of the method to distinguish among all four 6HNK stereoisomers (Figure 4C). The migration times for (2*R*,6*R*)‐6HNK, (2*S*,6*S*)‐6HNK, (2*R*,6*S*)‐6HNK, and (2*S*,6*R*)‐6HNK were 67.2, 67.8, 69.9, and 70.8 min, respectively. *R* among the neighboring peaks was 0.80, 3.17, and 0.94. All four 6HNK stereoisomers showed strong antidepressant effects in humans and are important in drug design and development. An example is the development of the antidepressant (5*R*)‐methyl‐(2*R*,6*R*)‐HNK [8]. In fractions II and III, a metabolite with *m*/*z* = 254 was found that can correspond to HK or MNK. Schmitz et al. identified HK only in fraction IV, whereas the rare metabolite MNK was not reported [14, 43, 44].

## 4 CONCLUDING REMARKS

The implementation of the partial filling approach enabled the combination of the advantages of CE and MS for chiral separation and detection of KET and its metabolites. The use of the chiral selector and a filling of 75% resulted admittedly in run times that are 10 times longer compared to those of CE–UV methods but allow collecting information on migration times and fragmentation simultaneously for all possible analytes within one analytical run. This is of great advantage for the identification and analysis of drugs and metabolites with multiple chiral centers at variable positions such as ketamine and its hydroxylated metabolites. With the herein presented method, it was possible to detect stereoisomers of NK, DHNK, and HNK, as well as the rare and low abundant metabolites HK, MNK, and DHK.

## CONFLICT OF INTEREST

The authors have declared no conflict of interest.

## Data Availability

The data that support the findings of this study are available from the corresponding author upon reasonable request.
